# Genetic Variants in *TNFSF4* and *TNFSF8* Are Associated With the Risk of HCV Infection Among Chinese High-Risk Population

**DOI:** 10.3389/fgene.2021.630310

**Published:** 2021-03-25

**Authors:** Zuqiang Fu, Weihua Cai, Jianguo Shao, Hong Xue, Zhijun Ge, Haozhi Fan, Chen Dong, Chunhui Wang, Jinwei Zhang, Chao Shen, Yun Zhang, Peng Huang, Ming Yue

**Affiliations:** ^1^Department of Epidemiology, School of Public Health, Nanjing Medical University, Nanjing, China; ^2^Institute of Epidemiology and Microbiology, Eastern Theater Command Centers for Disease Control and Prevention, Nanjing, China; ^3^Department of Hepatobiliary Surgery, Third Affiliated Hospital of Nantong University, Nantong, China; ^4^Department of Gastroenterology, Third Affiliated Hospital of Nantong University, Nantong, China; ^5^Department of Infectious Diseases, Third Affiliated Hospital of Nantong University, Nantong, China; ^6^Department of Critical Care Medicine, The Affiliated Yixing Hospital of Jiangsu University, Yixing, China; ^7^Department of Information, The First Affiliated Hospital of Nanjing Medical University, Nanjing, China; ^8^Department of Epidemiology and Statistics, School of Public Health, Soochow University Medical College, Suzhou, China; ^9^Center for Global Health, School of Public Health, Nanjing Medical University, Nanjing, China; ^10^Department of Anesthesiology, Affiliated Drum-Tower Hospital of Medical College of Nanjing University, Nanjing, China; ^11^Department of Infectious Diseases, The First Affiliated Hospital of Nanjing Medical University, Nanjing, China

**Keywords:** tumor necrosis factor superfamily, tumor necrosis factor receptor superfamily, hepatitis C virus, single nucleotide polymorphisms (SNPs), correlation analysis

## Abstract

**Background:**

The tumor necrosis factor superfamily (*TNFSF*) and TNF receptor superfamily (*TNFRSF*) play important roles in the immune responses to infections. The aim of this study was to determine the impact of single nucleotide polymorphisms (SNPs) of several *TNFSF/TNFRSF* genes on the risk of hepatitis C virus (HCV) infection in the Chinese high-risk population.

**Methods:**

The *TNFSF4*-rs1234313, *TNFSF4*-rs7514229, *TNFSF8*-rs3181366, *TNFSF8*-rs2295800, *TNFRSF8*-rs2298209, and *TNFRSF8*-rs2230625 SNPs were genotyped in 2309 uninfected controls, 597 subjects with spontaneous HCV clearance and 784 patients with persistent HCV infection using the TaqMan-MGB assay. The putative functions of the positive SNPs were determined using online bioinformatics tools.

**Results:**

After adjusting for gender, age, high-risk population, alanine transaminase (ALT), aspartate aminotransferase (AST), *IL28B*-rs12979860 and rs8099917 genotypes, the non-conditional logistic regression showed that rs7514229-T, rs3181366-T, and rs2295800-C were associated with an increased risk of HCV infection (all *P_*FDR*_* < 0.05). Combined analysis of rs7514229-T and rs3181366-T risk alleles showed that the subjects carrying 2–4 risk alleles were more susceptible to HCV infection compared with those lacking any risk allele (all *P* < 0.001). Furthermore, the risk of HCV infection increased with the number of risk alleles (*P_*trend*_* < 0.001). *In silico* analysis showed that rs7514229, rs3181366, and rs2295800 polymorphisms may affect the transcription of mRNA by regulating miRNA binding, TF binding, and promoter activation, respectively, which may have biological consequences.

**Conclusion:**

*TNFSF4*-rs7514229, *TNFSF8*-rs3181366, and *TNFSF8*-rs2295800 are associated with increased risk of HCV infection in the Chinese high-risk population.

## Introduction

Hepatitis C is the result of hepatitis C virus (HCV) infection and currently afflicts around 185 million people worldwide, of which 71 million are chronically infected (WHO, 2018; Spearman et al., 2019). Approximately 55–85% of the infected patients subsequently develop chronic hepatitis C (CHC) due to lack of viral clearance, which can lead to decompensated cirrhosis, hepatocellular carcinoma (HCC), and even death (Chinese Society of Hepatology and Chinese Society of Infectious Disease, 2019). WHO and other international organizations have pledged to eliminate HCV infection by 2030, which is incumbent on the development of effective vaccines and therapeutics (WHO, 2018). Although direct-acting antiviral drugs (DAAs) have been effective against HCV, only 1.3% of the patients in China have received these drugs owing to their high costs (Wei et al., 2016; Li and Chung, 2019). Furthermore, the attempts to develop an HCV vaccine have been largely unsuccessful due to the high genetic variability of this virus. Therefore, the molecular mechanisms underlying HCV infection, especially virus–host interactions, need further elucidation to circumvent the aforementioned limitations (Heim et al., 2016).

Studies show that genetic factors are a major determinant of the host response to HCV infection (Matsuura and Tanaka, 2018). Genetic variants of immune-related genes such as *IL28B* (Thomas et al., 2009), *HLA-DQB1* (Lee et al., 2018), and *IFN* (Welzel et al., 2009) have been implied in HCV infection. Tumor necrosis factor superfamily/tumor necrosis factor receptor superfamily (TNFSF/TNFRSF) proteins are expressed in immune cells, and are frequently activated or dysregulated in inflammatory diseases such as inflammatory bowel disease (IBD; De Voogd et al., 2016), systemic lupus erythematosus (SLE; Jackson and Davidson, 2019), rheumatoid arthritis (RA; Croft and Siegel, 2017), Crohn’s disease (CD; Hong et al., 2016), and hepatitis (Shin et al., 2016). Furthermore, genetic variants of *TNFRSF1A* (Yue et al., 2020), *TNFRSF5* (Tian et al., 2019), *TNFSF6* (Huang et al., 2020), and *TNFRSF11B* (Wu et al., 2019) influence the immune response to HCV infection. TNFSF4, TNFSF8, and their respective receptors also mediate the immune response in the manner similar to of sOX40L and sCD30L (Späth et al., 2017; Qin et al., 2018). However, it is still unclear whether polymorphisms in *TNFSF4/TNFRSF4* and *TNFSF8/TNFRSF8* have an effect on the host response to HCV infection.

In this study, we screened six single nucleotide polymorphisms (SNPs) of *TNFSF4/TNFRSF4* and *TNFSF8/TNFRSF8* in a Chinese cohort at high risk of HCV infection to determine their potential role in both HCV infection and CHC.

## Materials and Methods

### Study Population

A total of 3,976 subjects were recruited, including 816 hemodialysis (HD) patients from nine hospitals across southern China, 1,848 paid blood donors (PBD) from 20 villages within the Jiangsu Province, and 1312 people who use drugs (PWUD) from detoxification centers in Nanjing and Yixing City. In the 1990s, due to China’s poor medical level, HDs were usually infected with HCV through blood transmission. Besides, from 1980 to 1990, PBDs were monetarily compensated, and subsequently numerous donors were found to be infected with HCV. The plasma of some paid donors was separated and collected by plasmapheresis, and the other blood components that contained cross-contamination were returned to the donor. For PWUD, it was more likely to be the cross-use of contaminated needles and unsafe sex. The most common modes of transmission of HCV infection are blood transmission, sexual transmission, and mother-to-child transmission. As a result, we recruited HD, PBD, and PWUD as our research objects.

**FIGURE 1:**
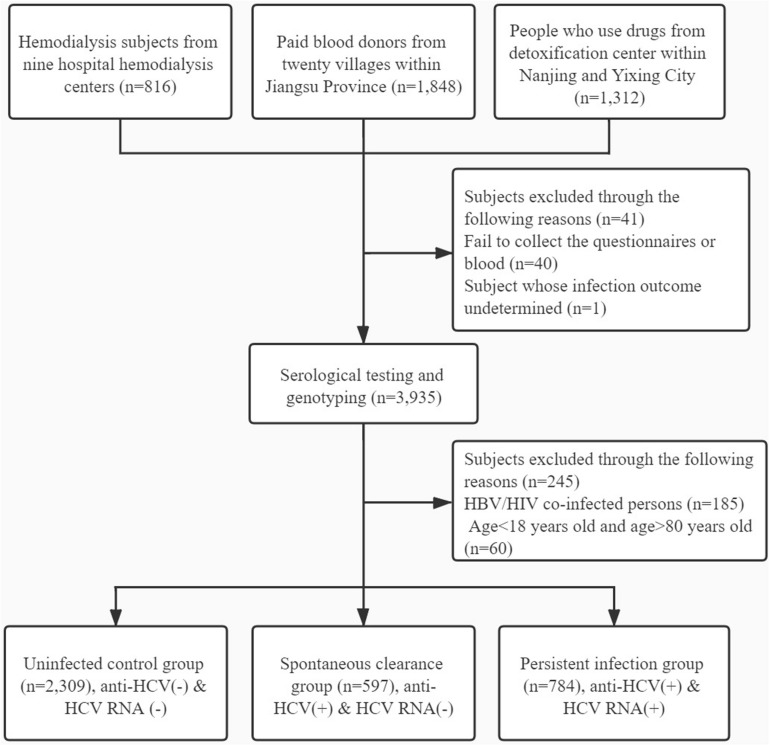
Flow diagram for inclusion of participants.

Participants were excluded for the following reasons: (1) failing to collect the blood or whose infection outcome undetermined; (2) HBV or HIV co-infection; (3) history of antiretroviral therapy; (4) age < 18 years and age > 80 years; (5) liver cirrhosis and other liver diseases; and (6) history of cancer or malignant tumor. The detailed flow diagram for recruiting participants is shown in Figure 1.

According to the diagnosis criteria of the “Guidelines for the prevention and treatment of hepatitis C (2019 version) (Chinese Society of Hepatology and Chinese Society of Infectious Disease, 2019),” the subjects were divided into the following groups: (1) uninfected controls—anti-HCV seronegative and HCV RNA seronegative, (2) spontaneous clearance—anti-HCV seropositive and HCV RNA seronegative, and (3) persistent infection—anti-HCV seropositive and HCV RNA seropositive. In addition, the spontaneous clearance and persistent infection groups were classified as HCV-infected.

### Serological Testing

After individual surveys using questionnaires, 5 ml venous blood was collected from each subject for further testing. HCV antibodies and RNA load were detected within 4 h, and the remaining blood was used for DNA extraction. The presence of anti-HCV antibody (anti-HCV) was tested by ELISA (Shanghai Abbott Laboratories, Shanghai, China), and HCV-RNA was isolated using Trizol LS reagent (Takara Biotech, Tokyo, Japan). Genomic DNA of leukocytes was extracted by the standard phenolic chloroform extraction method involving proteinase K digestion, phenol-chloroform purification, and ethanol precipitation. All DNA samples were stored at −20°C.

### SNP Selection and Genotyping Assays

The linkage disequilibrium (LD) data of the HapMap Phase II CHB (Chinese in Beijing) obtained from the 1000 Genomes Project database^1^ was imported into the Haploview software (version 4.2; Broad Institute, Cambridge, MA, United States). The candidate SNPs were screened with minor allele frequency (MAF) ≥ 0.05 and correlation coefficient *r*^2^ ≥ 0.8 as the thresholds. In addition, the sequences 2,000 bp upstream and downstream of the transcription initiation sites of *TNFSF4/TNFRSF4* and *TNFSF8/TNFRSF8* genes were also included in the analysis. The putative functional SNPs were then further screened using the RegulomeDB database^2^ and UCSC Genome Browser.^3^ Previously published SNPs associated with immunological or infectious diseases were also retrieved. Six SNPs (*TNFSF4*-rs1234313, rs7514229; *TNFSF8*-rs3181366, rs2295800; *TNFRSF4*-rs2298209; and *TNFRSF8*-rs2230625) were finally selected for further analysis. The candidate SNPs were genotyped using TaqMan real-time PCR assay in the LightCycler 480 II Real-Time PCR System (Roche Diagnostics, Mannheim, Germany). The primer and probe sequences are shown in Supplementary Table 1. The reaction parameters consisted of preheating at 50°C for 2 min and pre-denaturation at 95°C for 10 min, followed by 45 cycles of denaturation at 95°C for 15 s and annealing, and extension at 60°C for 1 min. The success rate for each SNP was above 95%. The experiment was repeated with randomly selected 10% of the samples, with a consistency rate of 100%. Genotyping was performed in a manner blinded to the clinical data, and two blank controls were set up for each 384-well format for quality control.

### *In silico* Analysis

The SNPs were functionally annotated using the SNPinfo website.^4^ The RegulomeDB online database^5^ was used to determine the regulatory role of the SNPs on the basis of RegulomeDB scores (Supplementary Table 2). The RNA Web Servers^6^ based on the latest Vienna RNA Package (version 2.4.16) was used to predict the secondary structures of single stranded RNA sequences and obtaining the minimum free energy (MFE), and the potential biological function was annotated using the UCSC Genome Bioinformatics website.^7^ The H3K27Ac histone marker data of seven cell lines (GM12878, H1-hESC, HSMM, HUVEC, K562, NHEK, and NHLF) was also analyzed.

### Statistical Analysis

Deviations from Hardy–Weinberg equilibrium (HWE) for each SNP among the controls were analyzed with χ^2^ test. Differences in demographic characteristics were described by mean ± SD or count (proportion) and compared by one-way ANOVA (for continuous variables) or the χ*^2^* test (for categorical variables). The association of the selected SNPs with HCV susceptibility and outcomes was estimated by constructing logistic regression models with age, gender, high-risk population, ALT, AST, *IL28B-*rs12979860, and *IL28B-*rs8099917 and each SNP as the covariates. The ORs with 95% CIs were calculated using co-dominant model, dominant model, recessive model, and additive model. All statistical analyses were performed by STATA 15.0 software (STATA Corp, College Station, TX, United States), and *P* < 0.05 was considered statistically significant. False discovery rate (FDR) correction was used to analyze the genotype distribution among the different groups.

## Results

### Basic Characteristics of Participants

As shown in Table 1, there were no significant differences in the distribution of the *IL28B*-rs12979860 genotypes among the three groups. In contrast, age, gender, ALT level, AST level, high-risk population, HCV genotype, and *IL28B*-rs8099917 (all *P* < 0.001) showed significant differences. The allele frequencies of five SNPs in the healthy uninfected controls were in accordance with the HWE (all *P* > 0.05), and only rs2298209 showed deviation (Supplementary Table 1).

**TABLE 1:** Demographical and clinical characteristics of the HCV control, spontaneous clearance, and persistent infection populations.

Variables	Uninfected control group (%)	Spontaneous clearance group (%)	Persistent infection group (%)	*P*
			
	*n* = 2,309	*n* = 597	*n* = 784	
Age (years, mean ± SD)	50.36 ± 14.72	49.52 ± 13.49	51.85 ± 12.10	**0.006^a^**
<50	1,114 (48,325)	268 (44.89)	314 (40.05)	
≥50	1,195 (51.75)	329 (55.11)	470 (59.95)	
Gender				**<0.001^b^**
Male	1,325 (57.38)	268 (44.89)	277 (35.33)	
Female	984 (42.62)	329 (55.11)	507 (64.67)	
ALT (U/L, median (IQR))	17.0 (11.0–25.0)	24.0 (15.0–40.5)	34.0 (21.0–59.0)	**<0.001^c^**
				**<0.001^b^**
≤40	2,109 (92.06)	447 (75.00)	465 (59.46)	
>40	182 (7.94)	149 (25.00)	317 (40.54)	
AST(U/L, median (IQR))	20.0 (16.0–25.0)	26.0 (19.0–35.0)	34.0 (24.0–53.0)	**<0.001^c^**
				**<0.001^b^**
≤40	2,197 (95.94)	467 (79.42)	479 (61.97)	
>40	93 (4.06)	121 (20.58)	294 (38.03)	
High-risk population				**<0.001^b^**
HD	581 (25.16)	92 (15.41)	76 (9.69)	
PBD	931 (40.32)	290 (48.58)	567 (72.32)	
PWUD	797 (34.52)	215 (36.01)	141 (17.98)	
HCV genotype, n (%)				**<0.001^b^**
1	–	43 (26.71)	479 (61.97)	
Non-1	–	121 (20.58)	294 (38.03)	
*IL28B-*rs12979860 (C > T)				0.166^b^
CC	1,989 (86.29)	526 (88.70)	690 (88.24)	
CT/TT	316 (13.71)	67 (11.30)	92 (11.76)	
*IL28B-*rs8099917 (T > G)				**<0.001^b^**
TT	1,788 (79.86)	518 (86.77)	686 (87.95)	
TG/GG	451 (20.14)	79 (13.23)	94 (12.05)	

*ALT, alanine transaminase; AST, aspartate aminotransferase; HCV, hepatitis C virus; HD, hemodialysis; PBD, paid blood donors; PWUD, people who use drugs.*

*^*a*^*P* value of one-way ANOVA among three groups.*

*^*b*^*P* value of χ^2^ test among three groups.*

*^*c*^One-way Kruskal–Wallis test among three groups.*

*Bold type indicates statistically significant results.*

### Association Between Selected Gene Polymorphisms and HCV Infection Outcomes

To determine the association between the SNPs and the risk of HCV infection, the patients with natural clearance and persistent infection were grouped as HCV-infected and compared with the uninfected group. After adjusting for gender, age, high-risk population, ALT, AST, *IL28B*-rs12979860, and *IL28B-*rs8099917, the different genetic models showed that *TNFSF4-*rs7514229 (dominant model: adjusted OR 1.37, 95% CI 1.13–1.66, *P* = 0.001; recessive model: adjusted OR 6.40, 95% CI 3.70–11.05, *P* < 0.001; additive model: adjusted OR 1.52, 95% CI 1.29–1.78, *P* < 0.001), *TNFSF8-*rs3181366 (dominant model: adjusted OR 1.20, 95% CI 1.03–1.40, *P* = 0.018; recessive model: adjusted OR 1.50, 95% CI 1.15–1.97, *P* = 0.003; additive model: adjusted OR 1.21, 95% CI 1.07–1.36, *P* = 0.002) and *TNFSF8-*rs2295800 (dominant model: adjusted OR 1.22, 95% CI 1.04–1.42, *P* = 0.012) were significantly associated with the susceptibility to HCV infection, while no significant correlation was seen between *TNFSF4-*rs1234313, *TNFRSF8-*rs2230625, and the risk of HCV infection (Table 2). After correcting for multiple comparison, the difference between rs7514229-T, rs3181366-T, rs2295800-C, and HCV infection were still significant (all *P_*FDR*_* < 0.05, Supplementary Table 3). In addition, there was no significant correlation between these SNPs and HCV infection chronicity when comparing the spontaneous clearance and persistent infection groups (all *P* > 0.05).

**TABLE 2:** Distribution of *TNFSF/TNFRSF* genes among the uninfected control, spontaneous clearance, and persistent infection groups.

SNPs (genotype)	Uninfected control group, n (%)	Spontaneous clearance group, n (%)	Persistent infection group, n (%)	OR (95% CI)^a^	*P*^a^	OR (95% CI)^b^	*P*^b^
*TNFSF4-*rs1234313					0.490*		0.416**
AA	978 (42.36)	270 (45.23)	333 (42.47)	1.00	–	1.00	–
AG	1,063 (46.04)	271 (45.39)	363 (46.30)	0.99 (0.84–1.16)	0.884	1.17 (0.92–1.48)	0.202
GG	268 (11.61)	56 (9.38)	88 (11.22)	0.89 (0.69–1.15)	0.375	1.35 (0.91–2.01)	0.131
Dominant model				0.97 (0.83–1.13)	0.679	1.20 (0.96–1.50)	0.116
Recessive model				0.90 (0.70–1.14)	0.375	1.25 (0.86–1.82)	0.239
Additive model				0.96 (0.86–1.07)	0.465	1.16 (0.98–1.38)	0.082
*TNFSF4-*rs7514229					<0.001*		0.621**
GG	1,821 (81.22)	464 (77.98)	612 (78.16)	1.00	–	1.00	–
GT	391 (17.44)	105 (17.65)	129 (16.48)	1.11 (0.91–1.36)	0.301	1.00 (0.74–1.36)	0.982
TT	30 (1.34)	26 (4.37)	42 (5.36)	**6.53 (3.77–11.3)**	**<0.001**	1.27 (0.74–2.17)	0.383
Dominant model				**1.37 (1.13–1.66)**	**0.001**	1.06 (0.80–1.39)	0.699
Recessive model				**6.40 (3.70–11.05)**	**<0.001**	1.27 (0.74–2.17)	0.381
Additive model				**1.52 (1.29–1.78)**	**<0.001**	1.07 (0.87–1.32)	0.519
*TNFSF8-*rs3181366					0.043*		0.801**
CC	1,239 (54.97)	302 (50.59)	403 (51.40)	1.00	–	1.00	–
CT	845 (37.49)	243 (40.70)	307 (39.16)	1.13 (0.96–1.33)	0.129	0.97 (0.77–1.23)	0.797
TT	170 (7.54)	52 (8.71)	74 (9.44)	**1.59 (1.20–2.10)**	**0.001**	1.28 (0.85–1.92)	0.241
Dominant model				**1.20 (1.03–1.40)**	**0.018**	1.02 (0.81–1.28)	0.864
Recessive model				**1.50 (1.15–1.97)**	**0.003**	1.29 (0.87–1.92)	0.201
Additive model				**1.21 (1.07–1.36)**	**0.002**	1.06 (0.90–1.26)	0.486
*TNFSF8-*rs2295800					0.073*		0.663**
TT	1,011 (43.79)	239 (40.03)	332 (42.35)	1.00	–	1.00	–
TC	1,035 (44.82)	298 (49.92)	373 (47.58)	**1.26 (1.07–1.48)**	**0.005**	0.94 (0.74–1.19)	0.607
CC	263 (11.39)	60 (10.05)	79 (10.08)	1.07 (0.82–1.38)	0.622	1.12 (0.75–1.67)	0.568
Dominant model				**1.22 (1.04–1.42)**	**0.012**	0.97 (0.77–1.22)	0.781
Recessive model				0.95 (0.71–1.21)	0.659	1.16 (0.80–1.69)	0.433
Additive model				1.10 (0.98–1.24)	0.093	1.01 (0.85–1.21)	0.878
*TNFRSF8-*rs223062*5*					0.728*		0.136**
AA	1,634 (70.77)	446 (74.71)	548 (69.90)	1.00	–	1.00	–
AG	619 (26.81)	139 (23.28)	215 (27.42)	0.94 (0.79–1.11)	0.452	1.23 (0.95–1.60)	0.112
GG	56 (2.43)	12 (2.01)	21 (2.68)	1.03 (0.63–1.70)	0.895	1.35 (0.64–2.85)	0.434
Dominant model				0.94 (0.80–1.12)	0.498	1.24 (0.97–1.60)	0.089
Recessive model				1.05 (0.64–1.72)	0.838	1.28 (0.61–2.70)	0.519
Additive model				0.96 (0.83–1.11)	0.591	1.21 (0.97–1.51)	0.091

*HCV, hepatitis C virus; SNP, single-nucleotide polymorphism; *TNFSF*, tumor necrosis factor superfamily; *TNFRSF*, tumor necrosis factor receptor superfamily.*

*A pair of alleles such as C/T, if C is a less frequent gene, then co-dominant model (TC vs. TT; CC vs. TT), dominant model (TC + CC vs. TT), recessive model (CC vs. TC + TT), and additive model (CC vs. TC vs. CC).*

*^*a*^The *P* value, OR, and 95% CIs of HCV-infected group (including HCV spontaneous clearance and persistent infection groups) vs. uninfected control group were calculated on the basis of the logistic regression model, adjusted by gender, age, high-risk population, ALT, AST, *IL28B*-rs12979860, and *IL28B-*rs8099917.*

^*b*^The *P* value, OR, and 95% CIs of (HCV persistent infection group *vs.* spontaneous clearance group) were calculated on the basis of the logistic regression model, adjusted by gender, age, high-risk population, ALT, AST, *IL28B-*rs12979860, and *IL28B-*rs8099917.

* The *P* value of χ^2^ test refers to the distribution of SNPs between HCV-infected group (including HCV spontaneous clearance and persistent infection groups) and uninfected control group.

** The *P* value of χ^2^ test refers to the distribution of SNPs between HCV spontaneous clearance and persistent infection groups.

*Bold type indicates statistically significant results.*

### Independent Analysis and Combined Analysis

To further analyze the combined effect of rs7514229, rs3181366, and rs2295800 on HCV infection, we performed independent tests on the three SNPs. As shown in Supplementary Table 4, after adjusting for rs3181366 (*P* = 0.189) or rs3181366 and rs7514229 (*P* = 0.371), the effect of rs2295800 was significantly reduced. Therefore, the combined effects of the risk alleles rs7514229-T and rs3181366-T were assessed. As shown in Table 3, the presence of 2–4 risk alleles were linked to an increased risk of HCV infection compared lack of any risk allele (all *P* < 0.001). Furthermore, the risk of HCV infection increased with the number of risk alleles (*P_*trend*_* < 0.001). Analysis of the combined risk genotypes (rs7514229-TT and rs3181366-TT) suggested that subjects with 1–2 risk genotypes were more susceptible (all *P* < 0.05), and both SNPs indicated a higher risk of HCV infection after the Cochran–Armitage trend test (*P_*trend*_* = 0.010) (Supplementary Table 5).

**TABLE 3:** The combined effects of risk alleles (rs7514229-T and rs3181366-T) on the risk of HCV infection.

Risk alleles^a^	Uninfected control group, *n* (%)	HCV-infected group, *n* (%)	HCV infection rate (%)	OR (95% CI)	*P*^b^
0	988 (45.18)	561 (40.71)	36.22	1.00	–
1	894 (40.88)	555 (40.28)	38.30	1.09 (0.92–1.29)	0.321
2	260 (11.89)	195 (14.15)	42.86	**1.48 (1.17–1.88)**	**0.001**
3–4	45 (2.06)	67 (4.86)	59.82	**3.68 (2.31–5.86)**	**<0.001**
Trend					**<0.001^c^**
0	988 (45.18)	561 (40.71)	36.22	1.00	–
1–4	1,198(54.82)	817 (59.29)	40.53	**1.25 (1.07–1.45)**	**0.005**

HCV, hepatitis C virus.

* HCV-infected group, HCV spontaneous clearance, and persistent infection groups.

*^*a*^Number of unfavorable alleles (rs7514229-T and rs3181366-T).*

*^*b*^*P* value was calculated by logistic regression analyses with adjustment for age, gender, high-risk population, ALT, AST, *IL28B*-rs12979860, and *IL28B*-rs8099917.*

*^*c*^*P* value for the Cochran–Armitage trend test.*

*Bold type indicates statistically significant results.*

### Stratified Analysis and Interaction Analysis of Positive SNPs

A stratified analysis of the positive SNPs was next performed in the age, gender, ALT level, AST level, and high-risk subgroups. The additive model showed that compared with the rs7514229-G allele, a significantly increased risk of HCV infection was attributed to rs7514229-T in the subgroups of age (all *P* < 0.05), female (adjusted OR 1.77, 95% CI 1.42–2.21, *P* < 0.001), ALT ≤ 40 U/L (adjusted OR 1.62, 95% CI 1.36–1.92, *P* < 0.001), AST ≤ 40 U/L (adjusted OR 1.45, 95% CI 1.23–1.72, *P* < 0.001), HD (adjusted OR 1.61, 95% CI 1.10–2.34, *P* = 0.013) and PBD (adjusted OR 1.57, 95% CI 1.25–1.99, *P* < 0.001) (Table 4). No significant heterogeneity was found among age, AST, and high-risk population stratifications (all *P* > 0.05), while the difference between gender (*P* = 0.028) and ALT level (*P* = 0.011) was evident (Supplementary Table 6). Furthermore, rs3181366-T was associated with a significantly increased risk of HCV infection in the subgroups of age <50 years (adjusted OR 1.34, 95% CI 1.12–1.61, *P* = 0.001), male (adjusted OR 1.31, 95% CI 1.10–1.56, *P* = 0.002), ALT ≤ 40 U/L (adjusted OR 1.19, 95% CI 1.05–1.35, *P* = 0.008), AST ≤ 40 U/L (adjusted OR 1.20, 95% CI 1.06–1.36, *P* = 0.003) and PWUD (adjusted OR 1.64, 95% CI 1.31–2.04, *P* < 0.001) as opposed to the rs3181366-C allele. No significant heterogeneity was found among age, gender, ALT, and AST stratifications (All *P* > 0.05), while that between high-risk population (*P* = 0.007) stratification was evident (Supplementary Table 7).

**TABLE 4:** Stratified analysis of rs7514229 and rs3181366 between HCV-infected and uninfected control groups.

Subgroups	rs7514229			rs3181366		
		
	OR (95% CI)^a^	*P*^a^	*P*^b^	OR (95% CI)^a^	*P*^a^	*P*^b^
Age			0.112			0.176
<50	**1.30 (1.03–1.66)**	**0.030**		**1.34 (1.12–1.61)**	**0.001**	
≥50	**1.70 (1.36–2.12)**	**<0.001**		1.13 (0.96–1.32)	0.135	
Gender			**0.028**			0.207
Male	1.21 (0.94–1.55)	0.134		**1.31 (1.10–1.56)**	**0.002**	
Female	**1.77 (1.42–2.21)**	**<0.001**		1.12 (0.95–1.32)	0.188	
ALT (U/L)			**0.011**			0.426
≤40	**1.62 (1.36–1.92)**	**<0.001**		**1.19 (1.05–1.35)**	**0.008**	
>40	0.96 (0.63-1.48)	0.869		1.40 (0.99–1.98)	0.057	
AST (U/L)			0.241			0.914
≤40	**1.45 (1.23–1.72)**	**<0.001**		**1.20 (1.06-1.36)**	**0.003**	
>40	2.53 (1.31–4.89)	0.950		1.23 (0.82–1.87)	0.318	
High-risk population			0.529			**0.007**
HD	**1.61 (1.10–2.34)**	**0.013**		1.27 (0.94–1.71)	0.114	
PBD	**1.57 (1.25–1.99)**	**<0.001**		1.01 (0.85–1.19)	0.933	
PWUD	1.29 (0.95–1.74)	0.101		**1.64 (1.31–2.04)**	**<0.001**	

*HCV, hepatitis C virus; ALT, alanine transaminase; AST, aspartate aminotransferase; HD, hemodialysis; PBD, paid blood donors; PWUD, people who use drugs.*

*^*a*^HCV-infected group (including HCV spontaneous clearance and persistent infection groups) vs. uninfected control group, deriving from four genetic statistical models of logistic regression analyses with adjustment for age, gender, high-risk population, ALT, AST, *IL28B*-rs12979860, and *IL28B*-rs8099917 (the stratified factor in each stratum was excluded).*

*^*b*^*P* value for the heterogeneity test.*

*Bold type indicates statistically significant results.*

To take into account the heterogeneity between SNPs and stratification, the interaction between the two SNPs and other factors in the context of HCV infection risk was analyzed (Table 5). Multiplicative interactions between rs7514229 genotypes and gender (*P_*interaction*_* = 0.034) were assessed, and a significantly higher risk of HCV infection was observed for females (all *P* < 0.001) and males with TT genotype (*P* < 0.001) compared with males with the GG genotype. For rs3181366 genotypes and high-risk population (*P_*interaction*_* = 0.016), the risk of HCV infection was significantly higher for PBD and PWUD compared with HD with CC genotype (all *P* < 0.05).

**TABLE 5:** Interaction analysis between two SNPs and other factors on the risk of HCV infection.

Genotypes	Variables	Uninfected control group, n (%)	HCV-infected group, n (%)*	OR (95% CI)	*P*^a^	*P*^b^
Rs7514229	Gender					**0.034**
GG	Male	1,049(57.61)	434 (40.33)	1.00	–	
GG	Female	772 (42.39)	642 (59.67)	**1.78 (1.48–2.13)**	**<0.001**	
GT	Male	239 (61.13)	90 (38.46)	0.94 (0.69–1.28)	0.700	
GT	Female	152 (38.87)	144 (61.54)	**2.28 (1.72–3.03)**	**<0.001**	
TT	Male	12 (40.00)	20 (29.41)	**4.72 (2.04–10.94)**	**<0.001**	
TT	Female	18 (60.00)	48 (70.59)	**14.72 (7.01–30.88)**	**<0.001**	
Rs3181366	High-risk population					**0.016**
CC	HD	314 (25.34)	84 (11.91)	1.00	–	
CC	PBD	467 (37.69)	447 (63.40)	**2.74 (2.02–3.73)**	**<0.001**	
CC	PWUD	458 (36.97)	174 (24.68)	**1.47 (1.03–2.09)**	**0.032**	
CT	HD	213 (25.21)	69 (12.55)	1.15 (0.78–1.71)	0.484	
CT	PBD	348 (41.18)	346 (62.91)	**2.94 (2.14–4.04)**	**<0.001**	
CT	PWUD	284 (33.61)	135 (24.55)	**1.83 (1.26–2.65)**	**0.002**	
TT	HD	39 (22.94)	15 (11.90)	1.76 (0.87–3.55)	0.116	
TT	PBD	99 (58.24)	47 (37.30)	**2.48 (1.58–3.89)**	**<0.001**	
TT	PWUD	32 (18.82)	64 (50.79)	**6.27 (3.58–10.99)**	**<0.001**	

HCV, hepatitis C virus; SNPs, single-nucleotide polymorphisms; ALT, alanine transaminase; HD, hemodialysis; PBD, paid blood donors; PWUD, people who use drugs.

* HCV-infected group, HCV spontaneous clearance and persistent infection groups.

*^*a*^*P* value was calculated by multiplicative model of logistic regression analyses with adjustment for age, gender, high-risk population, ALT, AST, *IL28B*-rs12979860, and *IL28B*-rs8099917 (the interaction item was excluded).*

*^*b*^*P* value for the interaction analysis.*

*Bold type indicates statistically significant results.*

### *In silico* Analysis of Positive SNP Function

**FIGURE 2:**
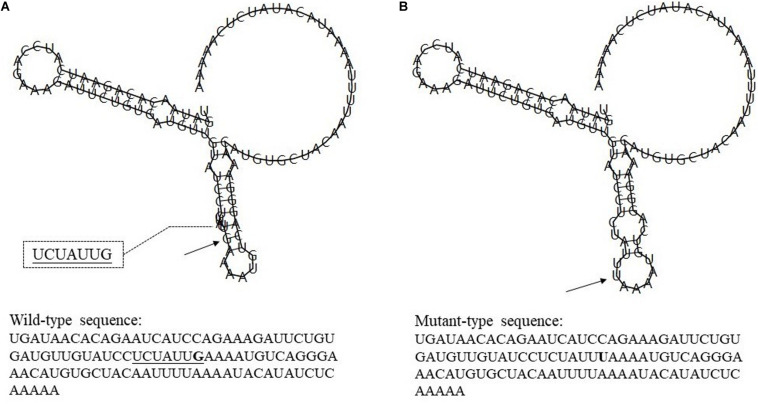
The influence of rs7514229 variants on *TNFSF4* mRNA centroid secondary structures. Changes in the local structure were illustrated by the RNAfold Web Server. The arrows indicate the location of the mutation (50 bases upstream and 50 bases downstream of the mutation) shown in bold type. The underlined sequence has overlapping nucleotide letters. *TNFSF4* rs7514229 changes the local structure and the minimum free energy of the mRNA centroid secondary structure (one with the smallest base pair distance) from −17.30 kcal/mol **(A)** to −16.60 kcal/mol **(B)**. The wild-type and mutant-type sequences are also listed (available at http://rna.tbi.univie.ac.at//cgi-bin/RNAWebSuite/RNAfold.cgi).

Rs7514229 is located in the three prime untranslated regions (3′UTR) of *TNFSF4*. Based on SNPinfo,^8^ rs7514229 is a putative microRNA-binding (miRNA-binding) site. To further analyze the effects of mutations on miRNA and transcriptional regulation, RNAfold^9^ was used to predict the secondary structure of mRNA and calculate the MFE of the centroid structure (one with minimal base pair distance). The secondary structure of the mRNA with mutant T allele differed from that of the wild G allele (Figure 2) and had a higher MFE (−16.60 kcal/mol vs. −17.30 kcal/mol).

**FIGURE 3:**
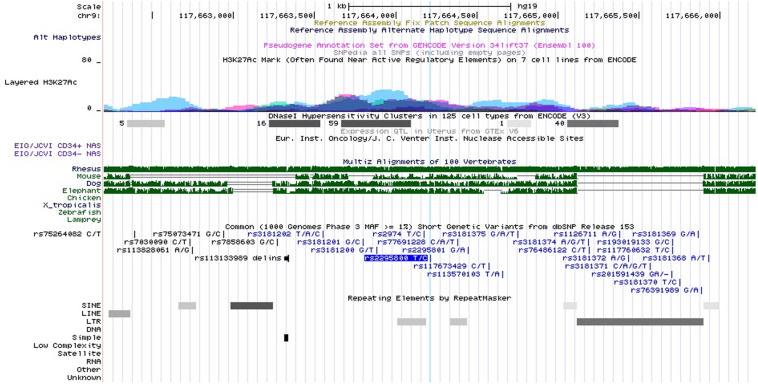
Functional annotation for rs2295800 using ENCODE data from UCSC genome browser. Transcription Factor ChIP-seq Clusters show binding regions of transcription factors and other regulatory proteins. Based on ENCODE project and the UCSC genome browser data, rs2295800 is located on the highest peak of the histone H3 acetylated lysine 27 (H3K27Ac) histone marker and this track shows enrichment of the H3K27Ac histone mark across the genome as determined by a ChIP-seq assay. The light blue line indicates the position of SNP rs2295800 (available at http://genome.ucsc.edu/).

Rs3181366 is located on the intron of *TNFSF8*. Its RegulomeDB score^10^ is 5, indicating potential functions like transcription factor (TF) binding or DNase peak (Supplementary Table 2). Rs2295800 is also located on the intron of *TNFSF8*, and its RegulomeDB score of 5 is suggestive of similar regulatory functions (Supplementary Table 2). Based on ENCODE and UCSC genome browser (see footnote 7), we found that rs2295800 was located on the highest peak of the histone H3 acetylated lysine 27 (H3K27Ac) histone marker, which was confirmed by the enrichment of H3K27Ac via ChIP-seq assay (Figure 3). The acetylation of lysine 27 may enhance transcription by blocking the spread of the repressive methylated H3K27Me3. Thus, rs2295800 polymorphism may affect the transcription of mRNA by affecting promoter activation, which may translate to disease susceptibility.

## Discussion

Our results show that *TNFSF4* rs7514229-T, *TNFSF8* rs3181366-T, and *TNFSF8* rs2295800-C are associated with an increased risk of HCV infection in the Chinese high-risk population. Furthermore, the presence of both rs7514229 and rs3181366 is significantly linked to a higher risk of HCV infection, and the risk increases with the number of risk alleles or genotypes. *In silico* analysis further showed that rs7514229, rs3181366, and rs2295800 polymorphisms may affect the transcription of mRNA by affecting miRNA binding, TF binding, and promoter activation, respectively, and thus mediate disease susceptibility.

*TNFSF4*, also known as *OX40L*, could be capable of interacting with its receptor on the late proliferation and sustained activation of T lymphocytes by extending the half-life of the cytokine mRNA (Vogel et al., 2013). Previous studies have shown that genetic variants of *TNFSF4/TNFRSF4* are associated with immune disorders such as Behcet’s Disease (Lu et al., 2016), autoimmune thyroid diseases (AITDs; Song et al., 2016), and SLEs (Cortini et al., 2017). We found that *TNFSF4* rs7514229-T (the mutant allele) was linked to an increased risk of HCV infection, and this effect was more evident in the age, female, lower ALT level (≤ 40 U/L), lower AST level (≤ 40 U/L), HD, and PBD subgroups. In addition, compared with male with rs7514229 GG genotype, a significant increased risk of HCV infection was observed for those who are female with GT/TT. Generally, being female is regarded as the common protective factor for hepatitis C because of the estradiol-related, more effective immune response (Fish, 2008). Based on bioinformatics analysis, we hypothesized that rs7514229 polymorphism may affect mRNA transcription by affecting the binding of miRNA, resulting in structural changes in the former that may regulate disease susceptibility. This hypothesis will have to be validated with functional studies on cellular models.

*TNFSF8*, also known as *CD30L*, interacts with its receptor on effector or memory T helper cells following activation by neutrophils, CD4^+^ T, and antigen-presenting cells, eventually mediating inflammatory diseases like IBD (Sun et al., 2008), RA (Barbieri et al., 2015), and CD (Hong et al., 2016). Some studies have also reported an association between the rs3181366 polymorphism and lung cancer (Wei et al., 2011) and myeloma bone disease (Durie et al., 2009). We observed that the *TNFSF8* rs3181366-T mutant allele was linked to an elevated risk of HCV infection. Moreover, the effect of rs3181366-T was prominent in <50 years of age, male, lower ALT level (≤40 U/L), lower AST level (≤40 U/L), and PWUD subgroups. The mutant *TNFSF8* rs2295800-C allele was also identified as a susceptibility locus for HCV infection, especially in the >50 years of age, lower ALT level (≤40 U/L), and lower AST level (≤40 U/L) subgroups. Based on the *in silico* analysis, the rs3181366 polymorphism may affect mRNA transcription by affecting TF binding and inducing structural changes with biological consequences. Rs2295800 was located on the highest peak of the H3K27Ac histone marker and may enhance transcription by blocking the spread of the repressive histone mark H3K27Me3. Thus, rs2295800 likely affects promoter activation and transcription.

The rs7514229 and rs3181366 SNPs showed independent effects on the risk of HCV infection, and the combined analysis of the rs7514229-T and rs3181366-T risk alleles suggested that subjects carrying two or more risk alleles were more susceptible to HCV infection. Furthermore, the risk increased with the number of risk alleles. Therefore, we hypothesized that genetic variants of TNFSF4 and TNFSF8 may have synergistic effects during the course of HCV infection. Since TNFSF8, TNFSF4, and their receptors play key roles in the differentiation and expansion of Th17 cells (Sun et al., 2010; Zhang et al., 2010), rs7514229 and rs3181366 may influence the outcome of HCV infection by affecting the Th17 population.

Although the results of this study are reliable and representative due to the reasonable design and large sample size, we must acknowledge some potential limitations. First, only six SNPs were selected and genotyped, which may be insufficient to fully analyze the relationship between *TNFSF4* and *TNFSF8* polymorphisms and HCV infection outcomes. However, the selection of candidate SNPs was based on strict and reasonable criteria. Also, this study was indeed a continuation of the later studies; the differences were the increase of the sample size of the population and the change of the pathway genes, and that FDR correction was used to solve the multiple comparisons problem of multiple SNPs in this study. In addition, this study lacked information on the prevalence of HCV genotypes, which may also affect the outcomes of HCV infection. Nevertheless, a previous study reported that HCV genotype 1 was the most common genotype in the Chinese population (Chen et al., 2017). Finally, the predicted biological functions of SNPs will need experimental validation.

Taken together, *TNFSF4* rs7514229-T, *TNFSF8* rs3181366-T, and *TNFSF8*-2295800-C are linked to an increased risk of HCV infection among the Chinese high-risk population. Our findings provide new insights into HCV screening and prevention, as well as vaccine development.

## Data Availability Statement

The datasets presented in this article are not readily available because the principle of confidentiality and Chinese relevant policies. Requests to access the datasets should be directed to Department of Science and Technology, Nanjing Medical University, kejichu@njmu.edu.cn.

## Ethics Statement

The studies involving human participants were reviewed and approved by Ethics Committee of the Eastern Theater Command Centers for Disease Control and Prevention, Nanjing, China. The patients/participants provided their written informed consent to participate in this study.

## Author Contributions

ZF, WC, PH, and MY designed and organized the study and supervised the whole project. ZF, JS, HX, ZG, CD, and JZ contributed to field survey, data collection, laboratory detection, and quality control. ZF, HF, CS, and PH performed the data cleansing and statistical analysis. WC, CW, YZ, and MY provided analysis tools and performed data interpretation. ZF, WC, PH, and MY wrote and critically revised the manuscript. All authors made substantial contributions to editing and drafting of the manuscript and read and approved the final manuscript.

## Conflict of Interest

The authors declare that the research was conducted in the absence of any commercial or financial relationships that could be construed as a potential conflict of interest.

## Footnotes

1 http://www.1000genomes.org/

2 http://www.regulomedb.org/

3 https://genome.ucsc.edu/

4 https://snpinfo.niehs.nih.gov/cgi-bin/snpinfo/snpfunc.cgi

5 http://www.regulomedb.org/

6 http://rna.tbi.univie.ac.at/cgi-bin/RNAWebSuite/RNAfold.cgi

7 http://genome.ucsc.edu/

8 https://snpinfo.niehs.nih.gov/cgi-bin/snpinfo/snpfunc.cgi

9 http://rna.tbi.univie.ac.at//cgi-bin/RNAWebSuite/RNAfold.cgi

10 https://www.regulomedb.org/regulome-search/

## Supplementary Material

Supplementary Table 1 Sequences of probes and primers specific for the *TNFSF4/TNFRSF4*, *TNFSF8/TNFRSF8*, and *IL28B* SNPs.

Supplementary Table 2 RegulomeDB scores.

Supplementary Table 3 Association between the *TNFSF/TNFRSF* SNPs and HCV infection outcomes in the co-dominant, dominant, recessive, and additive models of multivariable analyses.

Supplementary Table 4 Independent analysis of SNPs associated with the risk of HCV infection.

Supplementary Table 5 The combined effects of risk genotypes (rs7514229-TT and rs3181366-TT) on the risk of HCV infection.

Supplementary Table 6 Stratified analyses of the association between rs7514229 and the risk of HCV infection.

Supplementary Table 7 Stratified analyses of the association between rs3181366 and the risk of HCV infection.
