# Supplementary material for: Genetic Variants in TNFSF4 and TNFSF8 Are Associated With the Risk of HCV Infection Among Chinese High-Risk Population
Source: Front Genet. 2021 Mar 25;12:630310. doi: 10.3389/fgene.2021.630310 (PMC8027328; doi:10.3389/fgene.2021.630310)
Supplement: Supplementary Table 1 — Sequences of probes and primers specific for the TNFSF4/TNFRSF4, TNFSF8/TNFRSF8, and IL28B SNPs. [file Table_1.DOCX]

**Table S1.** Sequences of probes and primers specific for the *TNFSF4/TNFRSF4*, *TNFSF8/TNFRSF8* and *IL28B* SNPs.

| SNPs (Allele) | Gene | Region | MAF^a/b^ | *P^C^* | TaqMan-MGB probe/primers sequences (5’-3’) |
| --- | --- | --- | --- | --- | --- |
| rs1234313 | *TNFSF4* | intron | 0.346/0.326 | 0.417 | Probe-G: FAM- TAACACTATACGTTGCT-MGB |
| (A>G) |  |  |  |  | Probe-A: VIC-TCAACACTATACATTGCT-MGB |
|  |  |  |  |  | Forward primer: ACCTTCATCAGCTACAAGATCATGTAA |
|  |  |  |  |  | Reverse primer: CTGTTGCTTCTTGACTTTTTAATAATGG |
| rs7514229 | *TNFSF4* | 3' UTR | 0.101/0.122 | 0.088 | Probe-C: FAM-TTCCCTGACATTTTCAATA-MGB |
| (G>T) |  |  |  |  | Probe-A: VIC-TGACATTTTAAATAGAGGATAC-MGB |
|  |  |  |  |  | Forward primer: TGTAACTTCTCTGTGCCAAACCTT |
|  |  |  |  |  | Reverse primer: CGAACAGCCCTCCACCTTT |
| rs2298209 | *TNFRSF4* | 3' UTR | 0.132/0.130 | <0.001 | Probe-G: FAM- TACGTAAGCAGAGAGC-MGB |
| (G>C) |  |  |  |  | Probe-C: VIC- ATACGTAAGCACAGAGC-MGB |
|  |  |  |  |  | Forward primer: CGTCTGCCAAGGTTTTTATTGTG |
|  |  |  |  |  | Reverse primer: GCCAACTCTGCACCGTTCTAG |
| rs3181366 | *TNFSF8* | intron | 0.263/0.330 | 0.121 | Probe-G: FAM-CTTATTACTCAAGGCACAGG-MGB |
| (C>T) |  |  |  |  | Probe-A: VIC-TTATTACTCAAGACACAGGTAT-MGB |
|  |  |  |  |  | Forward primer: TCTTCCATGTTACTCCCTCTAGAGTCT |
|  |  |  |  |  | Reverse primer: CCAGCTTTCAGCATTGAAGAAA |
| rs2295800 | *TNFSF8* | intron | 0.338/0.430 | 0.939 | Probe-G: FAM-AAGATTTAAAAGAGAAAAAG-MGB |
| (T>C) |  |  |  |  | Probe-A: VIC-AAGATTTAAAAGAAAAAAAG-MGB |
|  |  |  |  |  | Forward primer: GTAGGACTCCAGCAAAGTTTGTCA |
|  |  |  |  |  | Reverse primer: TCCATACCACCACTGCTGATTT |
| rs2230625 | *TNFRSF8* | missense | 0.158/0.144 | 0.772 | Probe-G: FAM-TGGTCGGCTCCGGC-MGB |
| (A>G) |  |  |  |  | Probe-A: VIC-TGGTCGGCTCCAGC-MGB |
|  |  |  |  |  | Forward primer: TTCTGGGTGATCCTGGTGTTG |
|  |  |  |  |  | Reverse primer: ACAGCCACTTACTCTGCCGAAT |
| rs12979860 | *IL28B* | intron | 0.072/0.035 | 0.122 | Probe-T: FAM-TCCCCGAAGGGTGA-MGB |
| (C>T) |  |  |  |  | Probe-C: VIC-CGAAGGCGCGAAC-MGB |
|  |  |  |  |  | Forward primer: TGCCTGTCGTGTACTGAACCA |
|  |  |  |  |  | Reverse primer: GAGCGCGGAGTGCAATTC |
| rs8099917 | *IL28B* | 5' UTR | 0.109/0.035 | 0.060 | Probe-T: FAM-TGAGCAATTTCACC-MGB |
| (T>G) |  |  |  |  | Probe-C: VIC-TGAGCAATGTCACCC-MGB |
|  |  |  |  |  | Forward primer: CAATTTGTCACTGTTCCTCCTTTTG |
|  |  |  |  |  | Reverse primer: TAAAGATGTGGGAGAATGCAAATGA |

Abbreviations: SNPs, single nucleotide polymorphisms; MAF, minor allele frequency; *TNFSF*, tumor necrosis factor superfamily; *TNFRSF*, tumor necrosis factor receptor superfamily.

^a^ Minor allele frequencies in controls group.

^b^ Minor allele frequencies from HapMap of Han Chinese in Beijing, China (CHB) or East Asia (EAS).(available at [https://www.ncbi.nlm.nih.gov/projects/SNP/snp](https://www.ncbi.nlm.nih.gov/projects/SNP/snp_ref.cgi?rs=2229094)).

^C^ *P*-value of Hardy-Weinberg equilibrium for SNPs among uninfected control.

**Table S2.** RegulomeDB scores.

| Score | Supporting data |
| --- | --- |
| 1a | eQTL + TF binding + matched TF motif + matched DNase Footprint + DNase peak |
| 1b | eQTL + TF binding + any motif + DNase Footprint + DNase peak |
| 1c | eQTL + TF binding + matched TF motif + DNase peak |
| 1d | eQTL + TF binding + any motif + DNase peak |
| 1e | eQTL + TF binding + matched TF motif |
| 1f | eQTL + TF binding / DNase peak |
| 2a | TF binding + matched TF motif + matched DNase Footprint + DNase peak |
| 2b | TF binding + any motif + DNase Footprint + DNase peak |
| 2c | TF binding + matched TF motif + DNase peak |
| 3a | TF binding + any motif + DNase peak |
| 3b | TF binding + matched TF motif |
| 4 | TF binding + DNase peak |
| 5 | TF binding or DNase peak |
| 6 | Motif hit |
| 7 | other |

eQTL: expression Quantitative Trait Loci; TF: transcription factor; DNase: deoxyribonuclease

**Table S3.** Association between the *TNFSF/TNFRSF* SNPs and HCV infection outcomes in the co-dominant, dominant, recessive and additive models of multivariable analyses.

| SNPs | Co-dominant model ^a^ | | | | Dominant model ^a^ | | Recessive model ^a^ | | Additive model ^a^ | | Co-dominant model ^b^ | | | | Dominant model ^b^ | | Recessive model ^b^ | | Additive model ^b^ | |
| --- | --- | --- | --- | --- | --- | --- | --- | --- | --- | --- | --- | --- | --- | --- | --- | --- | --- | --- | --- | --- |
|  | *P* | *P*_FDR_^*^ | *P* | *P*_FDR_^*^ | *P* | *P*_FDR_^*^ | *P* | *P*_FDR_^*^ | *P* | *P*_FDR_^*^ | *P* | *P*_FDR_^*^ | *P* | *P*_FDR_^*^ | *P* | *P*_FDR_^*^ | *P* | *P*_FDR_^*^ | *P* | *P*_FDR_^*^ |
| Rs1234313(A>G) | 0.884 | 0.884 | 0.375 | 0.625 | 0.679 | 0.679 | 0.375 | 0.625 | 0.465 | 0.581 | 0.202 | 0.505 | 0.131 | 0.543 | 0.116 | 0.290 | 0.239 | 0.519 | 0.082 | 0.228 |
| Rs7514229(G>T) | 0.301 | 0.502 | **0.001** | **0.003** | **0.001** | **0.005** | **0.001** | **0.005** | **0.001** | **0.005** | 0.982 | 0.952 | 0.383 | 0.543 | 0.699 | 0.864 | 0.381 | 0.519 | 0.519 | 0.649 |
| Rs3181366(C>T) | 0.129 | 0.323 | **0.001** | **0.003** | **0.018** | **0.030** | **0.003** | **0.008** | **0.002** | **0.005** | 0.797 | 0.952 | 0.241 | 0.543 | 0.864 | 0.864 | 0.201 | 0.519 | 0.486 | 0.649 |
| Rs2295800(C>T) | **0.005** | **0.025** | 0.622 | 0.778 | **0.012** | **0.030** | 0.659 | 0.824 | 0.093 | 0.155 | 0.607 | 0.952 | 0.568 | 0.568 | 0.781 | 0.864 | 0.433 | 0.519 | 0.878 | 0.878 |
| Rs2230625(T>C) | 0.452 | 0.565 | 0.895 | 0.895 | 0.498 | 0.623 | 0.838 | 0.838 | 0.591 | 0.591 | 0.112 | 0.505 | 0.434 | 0.543 | 0.089 | 0.290 | 0.519 | 0.519 | 0.091 | 0.228 |

*Abbreviations*: *TNFSF*, tumor necrosis factor superfamily; *TNFRSF*, tumor necrosis factor receptor superfamily; SNPs, single nucleotide polymorphisms; FDR, false discovery rate.

A pair of alleles such as C/T, if C is a less frequent gene, then co-dominant model (TC *vs.* TT; CC *vs.* TT), dominant model (TC+CC *vs.* TT), recessive model (CC *vs.* TC+TT), and additive model (TT *vs* TC *vs.* CC).

^a^ HCV-infected group (including HCV spontaneous clearance and persistent infection groups) *vs*. uninfected control group, deriving from four genetic statistical models of logistic regression analyses with adjustment for age, gender, high-risk population, ALT, AST, *IL28B*-rs12979860 and *IL28B*-rs8099917.

^b^ HCV persistent infection group *vs.* spontaneous clearance group , deriving from four genetic statistical models of logistic regression analyses with adjustment for age, gender, high-risk population, ALT, AST, *IL28B*-rs12979860 and *IL28B*-rs8099917.

^*^ Adjusted *P*-value using FDR for multiple testing correction.

Notes: Bold type indicates statistically significant results.

**Table S4.** Independent analysis of SNPs associated with the risk of HCV infection.

| SNPs | OR (95% CI)^a^ | *P^a^* | OR (95% CI)^b^ | *P^b^* | OR (95% CI)^c^ | *P^c^* |
| --- | --- | --- | --- | --- | --- | --- |
| rs7514229 | 1.48(1.26-1.74)^d^ | <0.001^d^ | 1.48(1.26-1.74) | <0.001 | 1.51(1.29-1.77) | <0.001 |
| rs3181366 | 1.22(1.08-1.38) | 0.001 | 1.20(1.01-1.42)^d^ | 0.036^d^ | 1.16(0.98-1.37) | 0.082 |
| rs2295800 | 1.20(1.03-1.40) | 0.021 | 1.14(0.94-1.40) | 0.189 | 1.10(0.90-1.34)^d^ | 0.371^d^ |

Abbreviations: HCV, hepatitis C virus; SNPs, single nucleotide polymorphisms; OR, odds ratio; 95% CI, 95% confidence interval.

^a^ HCV-infected group (including HCV spontaneous clearance and persistent infection groups) *vs.* uninfected control group, deriving from additive model of logistic regression analyses with adjustment for rs7514229, age, gender, high-risk population, ALT, AST, *IL28B*-rs12979860 and *IL28B*-rs8099917.

^b^ HCV-infected group (including HCV spontaneous clearance and persistent infection groups) *vs*. uninfected control group, deriving from additive model of logistic regression analyses with adjustment for rs3181366, age, gender, high-risk population, ALT, AST, *IL28B*-rs12979860 and *IL28B*-rs8099917.

^c^ HCV-infected group (including HCV spontaneous clearance and persistent infection groups) *vs*. uninfected control group, deriving from additive model of logistic regression analyses with adjustment for rs2295800, age, gender, high-risk population, ALT, AST, *IL28B*-rs12979860 and *IL28B*-rs8099917.

^d^ HCV-infected group (including HCV spontaneous clearance and persistent infection groups) *vs*. uninfected control group, deriving from additive model of logistic regression analyses with adjustment for rs7514229, rs3181366, rs2295800, age, gender, high-risk population, ALT, AST, *IL28B*-rs12979860 and *IL28B*-rs8099917 (the marked SNP was excluded).

**Table S5.** The combined effects of risk genotypes (rs7514229-TT and rs3181366-TT) on the risk of HCV infection.

| Risk genotypes ^a^ | Uninfected control group, n (%) | HCV-infected group, n (%) | HCV infection rate (%) | OR (95% CI) | *P^b^* |
| --- | --- | --- | --- | --- | --- |
| 0 | 1,015(45.03) | 564(40.84) | 35.72 | 1.00 | - |
| 1 | 1,012(44.90) | 656(47.50) | 39.33 | **1.18(1.00-1.39)** | **0.046** |
| 2 | 227(10.07) | 161(11.66) | 41.49 | **1.53(1.18-1.98)** | **0.001** |
| Trend |  |  |  |  | **0.010^c^** |
| 0 | 1,015(45.03) | 564(40.84) | 35.72 | 1.00 | - |
| 1-2 | 1,239(54.97) | 817(59.16) | 39.74 | **1.24(1.06-1.44)** | **0.007** |

Abbreviations: HCV, hepatitis C virus; OR, odds ratio; 95% CI, 95% confidence interval.

^*^ HCV-infected group, HCV spontaneous clearance and persistent infection groups

^a^ Number of unfavorable alleles (rs7514229-TT and rs3181366-TT).

^b^ *P*-value was calculated by logistic regression analyses with adjustment for age, gender, high-risk population, ALT, AST, *IL28B*-rs12979860 and *IL28B*-rs8099917.

^c^ *P*-value for the Cochran-Armitage trend test.

Notes: Bold type indicates statistically significant results.

**Table S6.** Stratified analyses of the association between rs7514229 and the risk of HCV infection.

| Subgroups | Uninfected control group | | | Spontaneous clearance group | | | Persistent infection group | | | OR (95% CI)^*^ | *P*^*^ | *P*^**^ |
| --- | --- | --- | --- | --- | --- | --- | --- | --- | --- | --- | --- | --- |
|  | GG, n (%) | GT, n (%) | TT, n (%) | GG, n (%) | GT, n (%) | TT, n (%) | GG, n (%) | GT, n (%) | TT, n (%) |  |  |  |
| Age |  |  |  |  |  |  |  |  |  |  |  | 0.112 |
| ≤50 | 859(79.2) | 212(19.6) | 13(1.2) | 204(76.4) | 51(19.1) | 12(4.5) | 250(79.6) | 55(17.5) | 9(2.9) | **1.30(1.03-1.66)** | **0.030** |  |
| >50 | 962(83.1) | 179(15.5) | 17(1.5) | 260(79.3) | 54(16.5) | 14(4.3) | 362(77.2) | 74(15.8) | 33(7.0) | **1.70(1.36-2.12)** | **<0.001** |  |
| Gender |  |  |  |  |  |  |  |  |  |  |  | **0.028** |
| Male | 1,049(80.7) | 239(18.4) | 12(0.9) | 208(77.9) | 49(18.4) | 10(3.8) | 226(81.6) | 41(14.8) | 10(3.6) | 1.21(0.94-1.55) | 0.134 |  |
| Female | 772(82.0) | 152(16.1) | 18(1.9) | 256(78.1) | 56(17.1) | 16(4.9) | 386(76.3) | 88(17.4) | 32(6.3) | **1.77(1.42-2.21)** | **<0.001** |  |
| ALT (U/L) |  |  |  |  |  |  |  |  |  |  |  | **0.011** |
| ≤40 | 1,672(81.6) | 347(16.9) | 29(1.4) | 347(77.8) | 78(17.5) | 21(4.7) | 353(75.9) | 83(17.9) | 29(6.2) | **1.62(1.36-1.92)** | **<0.001** |  |
| >40 | 138(77.5) | 39(21.9) | 1(0.6) | 116(78.4) | 27(18.2) | 5(3.4) | 258(81.7) | 45(14.2) | 13(4.1) | 0.96(0.63-1.48) | 0.869 |  |
| AST (U/L) |  |  |  |  |  |  |  |  |  |  |  | 0.241 |
| ≤40 | 1,730(81.1) | 374(17.5) | 30(1.4) | 367(78.8) | 80(17.2) | 19(4.1) | 371(77.5) | 84(17.5) | 24(5.0) | **1.45(1.23-1.72)** | **<0.001** |  |
| >40 | 79(87.8) | 11(12.2) | 0(0) | 88(73.3) | 25(20.8) | 7(5.8) | 234(79.9) | 41(14.0) | 18(6.1) | **2.53(1.31-4.89)** | **0.006** |  |
| High-risk population | |  |  |  |  |  |  |  |  |  |  | 0.529 |
| HD | 444(79.1) | 107(19.1) | 10(1.8) | 67(72.8) | 22(23.9) | 3(3.3) | 57(75.0) | 14(18.4) | 5(6.6) | **1.61(1.10-2.34)** | **0.013** |  |
| PBD | 751(83.7) | 131(14.6) | 15(1.7) | 234(81.3) | 42(14.6) | 12(4.2) | 445(78.6) | 85(15.0) | 36(6.4) | **1.57(1.25-1.99)** | **<0.001** |  |
| PWUD | 626(79.9) | 153(19.5) | 5(0.6) | 163(75.8) | 41(19.1) | 11(5.1) | 110(78.0) | 30(21.3) | 1(0.7) | 1.29(0.95-1.74) | 0.101 |  |

*Abbreviations:* HCV, hepatitis C virus; OR, odds ratio; 95% CI, 95% confidence interval; ALT, alanine transaminase; AST, aspartate aminotransferase; HD, hemodialysis; PBD, paid blood donors; PWUD, People who use drugs.

^*^ HCV-infected group (including HCV spontaneous clearance and persistent infection groups) *vs.* uninfected control group, deriving from additive model of logistic regression analyses with adjustment for age, gender, high-risk population, ALT, AST, *IL28B-*rs12979860 and *IL28B-*rs8099917 (the stratified factor in each stratum was excluded).

^**^ *P*-value for the heterogeneity test.

Notes: Bold type indicates statistically significant results.

**Table S7.** Stratified analyses of the association between rs3181366 and the risk of HCV infection.

| Subgroups | Uninfected control group | | | Spontaneous clearance group | | | Persistent infection group | | | OR (95% CI) ^*^ | *P*^*^ | *P*^**^ |
| --- | --- | --- | --- | --- | --- | --- | --- | --- | --- | --- | --- | --- |
|  | GG, n (%) | GT, n (%) | TT, n (%) | GG, n (%) | GT, n (%) | TT, n (%) | GG, n (%) | GT, n (%) | TT, n (%) |  |  |  |
| Age |  |  |  |  |  |  |  |  |  |  |  | 0.176 |
| ≤50 | 619(57.1) | 409(37.7) | 56(5.2) | 138(51.5) | 108(40.3) | 22(8.2) | 170(54.1) | 111(35.4) | 33(10.5) | **1.34(1.12-1.61)** | **0.001** |  |
| >50 | 620(53.0) | 436(37.3) | 114(9.7) | 164(49.9) | 135(41.0) | 30(9.1) | 233(49.6) | 196(41.7) | 41(8.7) | 1.13(0.96-1.32) | 0.135 |  |
| Gender |  |  |  |  |  |  |  |  |  |  |  | 0.207 |
| Male | 729(56.5) | 475(36.8) | 87(6.7) | 138(51.5) | 107(39.9) | 23(8.6) | 139(50.2) | 101(36.5) | 37(13.4) | **1.31(1.10-1.56)** | **0.002** |  |
| Female | 510(53.0) | 370(38.4) | 83(8.6) | 164(49.9) | 136(41.3) | 29(8.8) | 264(52.1) | 206(40.6) | 37(7.3) | 1.12(0.95-1.32) | 0.188 |  |
| ALT (U/L) |  |  |  |  |  |  |  |  |  |  |  | 0.426 |
| ≤40 | 1,115(54.2) | 783(38.1) | 159(7.7) | 222(49.7) | 182(40.7) | 43(9.6) | 236(50.8) | 180(38.7) | 49(10.5) | **1.19(1.05-1.35)** | **0.008** |  |
| >40 | 112(62.6) | 59(33.0) | 8(4.5) | 80(53.7) | 60(40.3) | 9(6.0) | 166(52.4) | 126(39.8) | 25(7.9) | 1.40(0.99-1.98) | 0.057 |  |
| AST (U/L) |  |  |  |  |  |  |  |  |  |  |  | 0.914 |
| ≤40 | 1,173(54.7) | 810(37.8) | 160(7.5) | 240(51.4) | 183(39.2) | 44(9.4) | 244(50.9) | 187(39.0) | 48(10.0) | **1.20(1.06-1.36)** | **0.003** |  |
| >40 | 52(56.5) | 32(34.8) | 8(8.7) | 59(48.8) | 55(45.5) | 7(5.8) | 152(51.7) | 117(39.8) | 25(8.5) | 1.23(0.82-1.87) | 0.318 |  |
| High-risk population | |  |  |  |  |  |  |  |  |  |  | **0.007** |
| HD | 314(55.5) | 213(37.6) | 39(6.9) | 45(48.9) | 41(44.6) | 6(6.5) | 39(51.3) | 28(36.8) | 9(11.8) | 1.27(0.94-1.71) | 0.114 |  |
| PBD | 467(51.1) | 348(38.1) | 99(10.8) | 150(51.7) | 116(40.0) | 24(8.3) | 297(52.4) | 230(40.6) | 40(7.1) | 1.01(0.85-1.19) | 0.933 |  |
| PWUD | 458(59.2) | 284(36.7) | 32(4.1) | 107(49.8) | 86(40.0) | 22(10.2) | 67(47.5) | 49(34.8) | 25(17.7) | **1.64(1.31-2.04)** | **<0.001** |  |

*Abbreviations:* HCV, hepatitis C virus; OR, odds ratio; 95% CI, 95% confidence interval; ALT, alanine transaminase; AST, aspartate aminotransferase; HD, hemodialysis; PBD, paid blood donors; PWUD, People who use drugs.

^*^ HCV-infected group (including HCV spontaneous clearance and persistent infection groups) *vs.* uninfected control group, deriving from additive model of logistic regression analyses with adjustment for age, gender, high-risk population, ALT, AST, *IL28B-*rs12979860 and *IL28B-*rs8099917 (the stratified factor in each stratum was excluded).

^**^ *P*-value for the heterogeneity test.

Notes: Bold type indicates statistically significant results.
